# Sex-biases in distribution and resource use at different spatial scales in a migratory shorebird

**DOI:** 10.1002/ece3.503

**Published:** 2013-03-09

**Authors:** José A Alves, Tómas G Gunnarsson, Peter M Potts, William J Sutherland, Jennifer A Gill

**Affiliations:** 1 School of Biological Sciences, University of East Anglia, Norwich Research ParkNorwich, NR4 7TJ, UK; 2 University of Iceland, South Iceland Research CentreTryggvagata 36, Selfoss, IS-800, Gunnarsholt, Hella, IS-851, Iceland; 3 Farlington Ringing Group, Solent Court CottageChilling Lane, Warsash, Southampton, SO31 9HF, UK; 4 Department of Zoology, Conservation Science Group, University of CambridgeDowning St, Cambridge, CB2 3EJ, UK

**Keywords:** Foraging, migratory range, non-breeding, segregation, sex dimorphism, waders

## Abstract

In migratory species, sexual size dimorphism can mean differing energetic requirements for males and females. Differences in the costs of migration and in the environmental conditions occurring throughout the range may therefore result in sex-biases in distribution and resource use at different spatial scales. In order to identify the scale at which sexual segregation operates, and thus the scale at which environmental changes may have sex-biased impacts, we use range-wide tracking of individually color-ringed Icelandic black-tailed godwits (*Limosa limosa islandica*) to quantify sexual segregation at scales ranging from the occupation of sites throughout the non-breeding range to within-site differences in distribution and resource use. Throughout the range of this migratory shorebird, there is no evidence of large-scale sex differences in distribution during the non-breeding season. However, the sexes differ in their selection of prey types and sizes, which results in small-scale sexual segregation within estuaries. The scale of sexual segregation therefore depends on the scale of variation in resource distribution, which, in this system, is primarily within estuaries. Sexual segregation in within-site distribution and resource use means that local-scale anthropogenic impacts on estuarine benthic prey communities may disproportionately affect the sexes in these migratory shorebirds.

## Introduction

Sexual size dimorphism is a widespread phenomenon across the animal kingdom (Darwin 1871). For dimorphic species, size differences between the sexes can create distinct energetic demands, resulting in different strategies and behaviors for each sex (Blanckenhorn 2005). The larger sex will require more food and energy (Blanckenhorn et al. 1995) and may experience higher mortality (Vollrath and Parker 1992; Nebel and Ydenberg 2005), but may also be more tolerant of extreme climatic conditions (McNab 1970). In migratory species, environmental conditions can differ greatly between the breeding and non-breeding seasons, and so sexual size dimorphism may result in males and females facing different trade-offs in migratory strategies and winter site selection.

Migratory shorebirds often range over large geographic areas within their annual cycle, and the variation in form and magnitude of sexual dimorphism found in this group encompasses virtually the entire range found in birds (Jehl and Murray 1986). Many species show a considerable degree of reverse size dimorphism, in which males are smaller than females (Jehl and Murray 1986; Székely et al. 2004), probably as a result of sexual selection (Székely et al. 2000) and the differing selection pressures influencing each sex during the breeding season. For example, although female preferences for intricate display flights (Andersson and Norberg 1981; Blomqvist et al. 1997a) may result in selection for smaller males, larger females often lay larger eggs (Thompson and Hale 1991) and chicks hatched from larger eggs can grow faster (Schifferli 1973; Ramos 2001) and have higher survival rates (Parsons 1970; Perrins 1996; Blomqvist et al. 1997b). As most shorebirds have a fixed clutch size of four eggs (Cramp and Simmons 1983), higher productivity cannot be achieved by laying larger clutches, which is likely to increase selection pressures on egg size, and consequently on female size.

Sexual size dimorphism is also likely to have implications during the non-breeding season. For instance, the larger sex may be better able to survive on colder sites (large-scale sexual segregation) (Choudhury and Black 1991; Gill et al. 1995; Cristol et al. 1999), or the sexes may winter in the same location, but forage on different types or sizes of prey (small-scale sexual segregation) (Summers et al. 1990; Durrell et al. 1993; Temeles et al. 2000; Zharikov and Skilleter 2002; Nebel and Thompson 2005). Large-scale sexual segregation may result in the sexes wintering in distinct parts of the range and experiencing different environmental conditions and migration distances, and consequently different energetic demands (Alves et al. 2012a, 2013). Similarly, the consequences of small-scale sex differences in foraging distribution or behavior will be influenced by relative prey profitability (Sutherland et al. 1996), even when energetic demands such as thermoregulation are similar for all individuals. Large-scale sexual segregation coupled with small-scale sex differences in resource availability has been described for the western sandpiper (*Calidris mauri*), in which larger billed females are commoner in the southern part of the range (Nebel 2005). Here, females are able to probe deeper into the sediment as estuarine prey buries deeply in these warmer locations (Nebel 2005; Nebel and Thompson 2005; Mathot et al. 2007). By contrast, small-scale sexual segregation has been reported in bar-tailed godwits (*Limosa lapponica*), with the larger females being proportionately more abundant in higher quality foraging patches within mudflats (Both et al. 2003). Thus, the distribution of the sexes can be influenced by prey distribution, but the energetic consequences of such segregation will depend on the relative profitability of different prey types and their abundance in different parts of the migratory range.

The Icelandic black-tailed godwit (*Limosa limosa islandica*) is a sexually dimorphic shorebird (Gunnarsson et al. 2006a) that breeds almost exclusively in Iceland and winters on western European coasts, from the United Kingdom and Ireland in the north, to the Iberian Peninsula in the south. Wintering over this latitudinal range results in individuals experiencing different migration distances (Alves et al. 2012a), energetic demands and prey resources (Alves et al. 2013). As female godwits are ca. 15–18% heavier than males, they will have higher food requirements and must use more energy than males to migrate the same distances (Alves et al. 2012a), which could result in large-scale sexual segregation. However, if sexual segregation is a result of sex differences in resource exploitation, then the scale of the segregation will depend on the scale of variation in resource abundance. Consequently, female and male godwits might differ in either their large-scale winter distribution and/or their small-scale use of foraging locations and prey types. In the breeding season, better quality sites tend to be inhabited by smaller males and there is evidence to suggest that larger females tend to be paired with smaller males (Gunnarsson et al. 2012). However, the consequences of variation in body size in the non-breeding season are unknown. Here, we use sightings of individually color-marked godwits and detailed observations of foraging godwits to assess the extent of sex differences in choice of (1) winter location throughout the range; (2) within-estuary foraging locations; and (3) within-site resource use. We conclude by discussing the consequences and conservation implications of sexual segregation for migratory shorebirds.

## Methods

### Large-scale sexual segregation in Icelandic godwits

Between 1993 and 2008, adult Icelandic godwits were captured, measured, and fitted with individual combinations of color-rings at locations across the range: south England (138 ringed since 1995 on the Solent, 50°50′N 0°55′W), east England (464 ringed since 1993 on either the Wash estuary, 52°56′N 0°06′W or Breydon Water, 52°36′N 1°42′W), Iceland (519 ringed at sites throughout the country since 1999), and west Portugal (166 ringed on the Tagus estuary, 38°44′N 8°59′W since 2006). Regular scanning of godwit flocks throughout the migratory range by a network of >2000 volunteer observers has resulted in >60% of all marked individuals being observed during winter months (Gunnarsson et al. 2006b). As adult godwits are long-lived and highly philopatric to both breeding and winter locations (Gill et al. 2002), any sightings between November and mid-February of any year were used to assign a region of winter location to these individuals.

Godwits were sexed using a discriminant function analysis of bill and wing measurements, which has previously been shown to correctly classify *c*. 97% of individuals (Gunnarsson et al. 2006a). Godwits for which a valid wing length was impossible to determine due to primary moult, and those within the overlap zone of the discriminant function, were conservatively sexed on the bill length measurement: male if below 88 mm (*n* = 77) or female if above 92 mm (*n* = 42) (Gunnarsson et al. 2006a).

To account for sex differences in capture probability in relation to location and time of year, sex ratio was estimated separately for individuals caught during three distinct periods of the annual cycle: (1) arrival in Iceland during April (total number of godwits = 375; Male to Female ratio at capture = 2.05), when males are likely to be proportionately more abundant due to pressure for early arrival on the breeding sites (Kokko et al. 2006); (2) during the breeding season in Iceland (*n* = 144; Male to Female ratio at capture = 1.12), when sex differences in nest attendance patterns can mean that one sex is likely to be caught more frequently; (3) during autumn migration and winter (*n* = 768; Male to Female ratio at capture = 1.39), when the sexes are less likely to differ in capture probability. Chi-squared goodness-of-fit tests were used to compare the observed number of color-ringed male godwits from each capture period wintering across the seven regions to the expected sex ratio (calculated from the proportion of males in that capture period).

### Small-scale sexual segregation on the Tagus estuary

Icelandic godwits foraging on estuarine flats on the Tagus estuary in Portugal, one of the key wintering site for this population (Alves et al. 2010), feed almost exclusively on bivalves (mostly *Scrobicularia plana*) and polychaetes (mostly *Hediste diversicolor*), although smaller items such as the gastropod *Hydrobia ulvae* are also occasionally consumed (ca. 6%, Moreira 1994). During the winters 2006–2007 and 2007–2008, the mudflats regularly used by wintering godwits on the Tagus estuary (Moreira 1993) were surveyed fortnightly to locate foraging flocks and color-ringed individuals. Average prey intake rates for each flock were estimated using the methods described in Gill et al. (2001a), in which randomly selected focal individuals are observed for the time taken to complete ten paces, during which the number, identity (polychaete, bivalve, or gastropod), and size of every prey item consumed are recorded (prey size classified as small: 3–5.5 mm bivalves; 3–9.9 mm polychaetes; 2–6 mm gastropods; medium: 5.6–9.5 mm bivalves; 10–19.9 mm polychaetes; large: 9.6–14.5 mm bivalves; 20–49.9 mm polychaetes; and very large: 14.6–20 mm bivalves; >50 mm polychaetes). Prey identity can be determined as there are conspicuous differences in godwit foraging behavior for different prey; godwits foraging on polychaetes peck deeply into the sediment (> 30% of bill length is inserted), whereas godwits foraging on bivalves use continuous shallow pecks (<20% of bill length is inserted) and godwits foraging on gastropods forage by stitching on the surface of the sediment. Flocks (*n* = 98) were classified as polychaete or bivalve-foraging, according to the prey type being consumed by the majority of focal individuals (no flock was ever recorded in which the majority of godwits were foraging on gastropods). In addition, the bill length and overall body size of each focal individual was used to assign a sex to each bird, including color-ringed individuals of known sex, which allowed estimating error rates in assigning sex during observations. Chi-squared tests of independence were used to test for differences in the number of male and female godwits (1) present in polychaete or bivalve-foraging flocks, and (2) foraging on polychaetes or bivalves within foraging flocks. Focal observations of prey intake rates of color-ringed individuals (*n* = 200 observations) for which bill length had been measured on capture (*n* = 40 individuals) were used to test for differences in prey size classes attained by godwits with differing bill lengths using Fisher's exact test of independence. In order to test for sex-specific differences in site use, the proportion of re-sightings of individually color-ringed male and female godwits on sites dominated by polychaetes was compared with a Mann–Whitney *U*-test. All observations were carried out by a single observer (JAA) using a ×20–60 telescope.

### Resource abundance and distribution on the Tagus estuary

**Figure 1:**
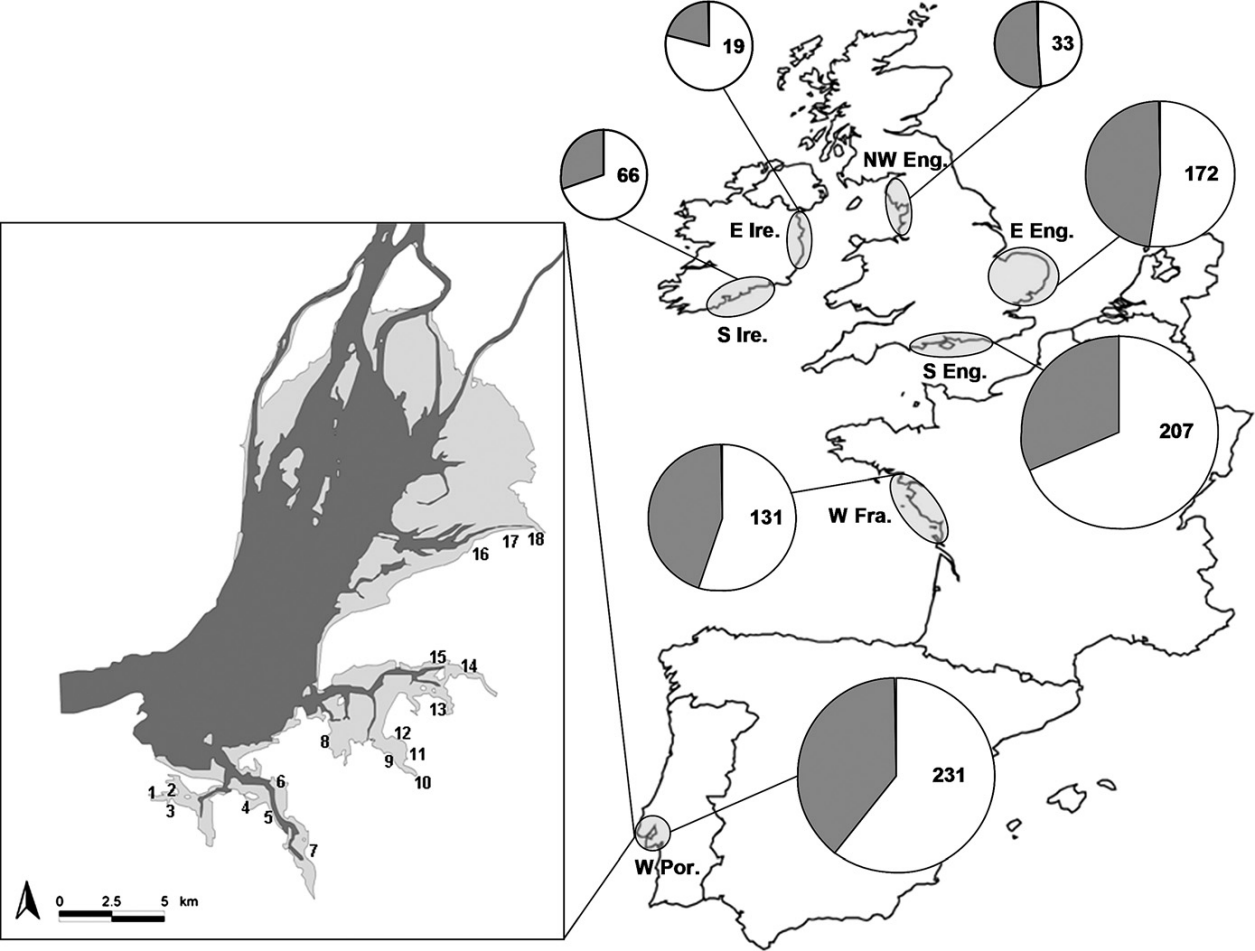
Proportions of wintering male (white) and female (gray) Icelandic black-tailed godwits in the seven main winter regions. Chart size is proportional to the number of marked individuals recorded in each site with numbers reported within each chart. Note that expected sex ratios are not equal (i.e., 1:1) and vary with the capture season and location of these individuals – see Methods for details. The inset shows the 18 sites at which godwit foraging behavior was recorded, and the six sites (3, 6, 8, 9, 13, and 18) at which prey were sampled, on the intertidal mudflats (light gray) of the Tagus estuary, West Portugal (dark gray indicates the area below mean low water).

Prey distribution and abundance was surveyed at six sites across the Tagus estuary (Fig. 1 inset), which are regularly used by foraging godwit flocks (Moreira 1993). From October 2006 to March 2007, these locations were visited monthly and 6–12 sediment cores were extracted at randomly located points within the area used by foraging godwits. Each sediment core was 9 cm in diameter and 15 cm deep, encompassing the maximum foraging depth of Icelandic godwits (Gill et al. 2001a). The sediment was sieved through a 1-mm mesh in situ, and all invertebrates were collected and taken to the laboratory for identification and measurement. Prey density for each site was calculated by dividing the total number of prey items in each core by the core area, and then averaged across the number of cores collected in each patch.

### Prey profitability

Focal observations of foraging godwits were used to calculate prey handling times for each size class of *S. plana* and *H. diversicolor*. Focal individuals were observed while probing the sediment and two stopwatches were started when a godwit detected a prey item and stopped probing. The first stopwatch was stopped when the prey item was extracted from the sediment, giving a measure of extraction time, and the second stopwatch was stopped when the prey item was swallowed (which ends with a conspicuous backward head movement) to give a measure of total handling time. Prey type and size class were recorded for all of these observations *(S. plana*, *n* = 112; *H. diversicolor*, *n* = 30). Differences in extraction and handling times between prey size classes were tested with one-way analysis of variance (ANOVA) and post-hoc tests. The ash-free dry mass content of the average prey size item within each category (reconstructed from fecal and prey samples, details in Alves et al. 2012b) was estimated using published formulae for those prey items, previously established for the Tagus estuary (Moreira 1994; Santos et al. 2005) and used to calculate intake rates (g/s, number of prey items of each size class consume per second multiplied by ash-free dry mass) and handling efficiency (g/s, average handling time of each size class multiplied by ash-free dry mass). Intake rates specific to each prey type and size were calculated by pooling observations in which the focal individual consumed only the same prey type and size during the observation period. Energetic intake rates (kJ/s) and prey energetic handling efficiency (kJ/s) were calculated by multiplying the mean intake rate and handling efficiency for each prey type and size class, respectively, by published estimates of the energetic content of *S. plana* (21.6 kJ/g) and *H. diversicolor* (22.0 kJ/g) (Zwarts and Wanink 1993).

## Results

### Large-scale sexual segregation in Icelandic godwits

Winter location was identified for the majority of godwits caught at all periods of the annual cycle and across the range: arrival in Iceland (60%); breeding in Iceland (71%); non-breeding (69%). Across the seven major wintering locations for Icelandic godwits, ranging from Britain and Ireland to Portugal, the observed number of males did not differ significantly from the expected proportion (calculated from the sex ratio at capture): arrival in Iceland (*χ*^2^_6_ = 4.44; *P* = 0.62); breeding in Iceland (*χ*^2^_6_ = 3.01; *P* = 0.81); and non-breeding season (*χ*^2^_6_ = 5.7; *P* = 0.46) (Fig. 1). Therefore, we found no evidence of strong geographic sexual segregation in wintering godwits.

### Resource abundance and distribution on the Tagus estuary

**Figure 2:**
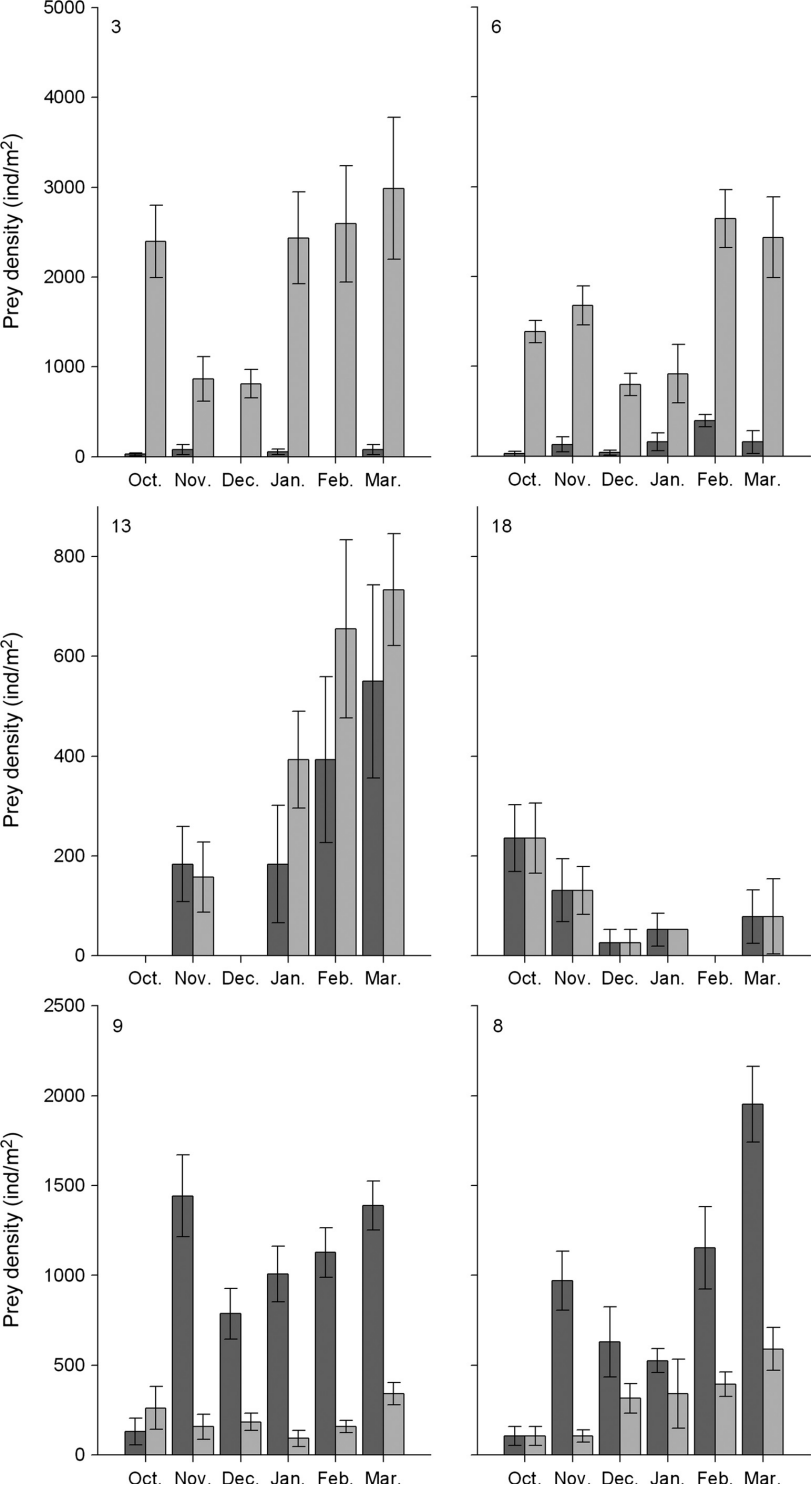
Densities (mean ± SE ind./m^2^) of *Scrobicularia plana* (dark gray bars) and *Hediste diversicolor* (light gray bars) at six godwit foraging locations on the Tagus estuary during each month of the winter 2006–2007. Site location is given by number (see Fig. 1 for location and Table 1 for flock diet composition of each site). Panels are displayed from top to bottom according to *Hediste diversicolor* density.

Prey species composition and abundance varied greatly across the six godwit foraging sites at which prey densities were measured on the Tagus estuary (Fig. 2). Two sites (3 and 6) had very high densities of *H. diversicolor*, two (8 and 9) had high densities of *S. plana* and the remaining two (13 and 18) comprised relatively low densities of both prey species. Accordingly, across the 18 sites at which foraging godwits were recorded, the proportion of godwits in each flock foraging on *S. plana* or *H. diversicolor* also ranges from the entire flock foraging on *S. plana* to more than 95% foraging on *H. diversicolor* (average number of flocks observed per site: 5.4 ± 0.8 SE) (Table 1).

**Table 1:** The proportion of focal godwits foraging on *Scrobicularia plana* or *Hediste diversicolor* in flocks at 18 sites across the Tagus estuary. Sites are ordered according to the proportion of godwits foraging on bivalves. See Fig. 1 for site locations. N indicates number of flocks sampled

Site	Diet of flocks				

	*S. plana*		*H. diversicolor*		*N*
	
	Mean	SE	Mean	SE	
12	100.0	0.0	0.0	0.0	4
15	100.0	0.0	0.0	0.0	6
16	100.0	0.0	0.0	0.0	1
17	100.0	0.0	0.0	0.0	3
11	96.4	3.6	0.0	0.0	5
18	96.2	1.8	3.3	1.5	7
13	94.8	1.7	3.4	3.4	2
9	92.0	8.0	1.0	1.0	4
14	82.7	4.6	4.1	3.6	14
10	67.2	8.6	28.9	10.6	3
4	61.8	15.8	21.5	16.5	3
5	38.1	17.6	60.8	18.0	3
1	37.5	13.9	45.6	11.5	6
8	31.4	14.9	67.6	15.5	7
3	24.9	5.4	65.0	7.7	7
2	13.4	8.3	86.6	8.3	3
6	3.6	2.2	93.6	2.8	12
7	2.1	1.7	96.1	1.9	8

### Small-scale sexual variation in resource use

**Figure 3:**
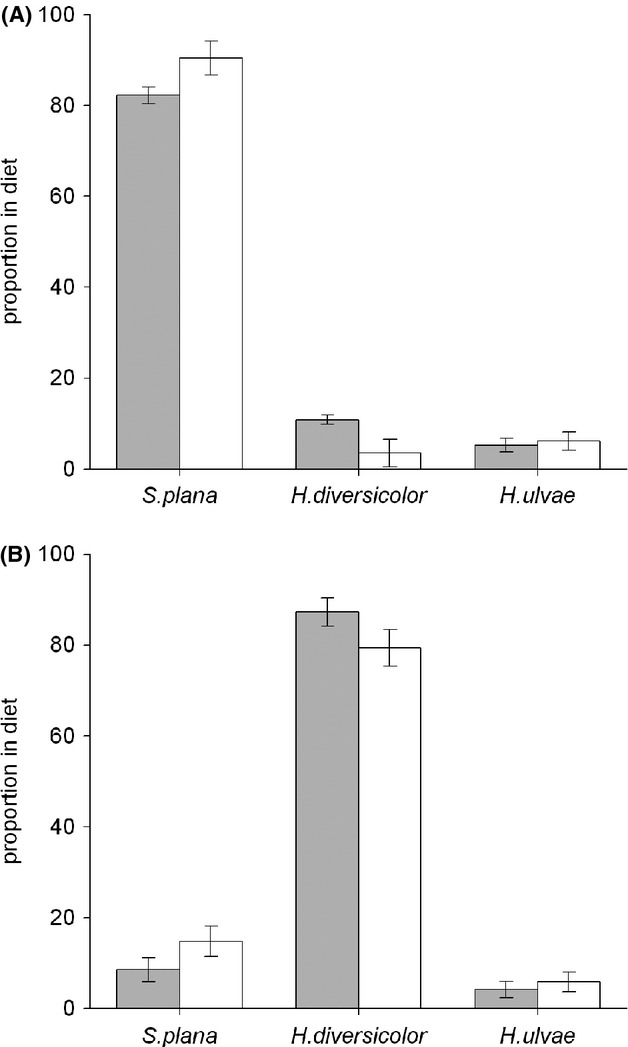
The mean (± SE) proportion of three different prey species in the diet of female (gray bars) and male (white bars) black-tailed godwits in flocks foraging primarily on (A) *Scrobicularia plana* or (B) *Hediste diversicolor*.

The number of male and female godwits in foraging flocks varied in relation to the dominant prey type being consumed, with the 57 flocks foraging on *S. plana* comprising an average of ca. 67.5% (± 1.7 SE) males, whereas flocks foraging on *H. diversicolor* comprised an average of ca. 52.5% (± 2.7 SE) females (*χ*^2^_1_ = 125.8, *P* < 0.001). The number of males and females foraging on different prey within these flocks also differed (*χ*^2^_1_ = 151.1, *P* < 0.001; Fig. 3). Approximately, 68% of all observed males (*n* = 1755) were foraging on *S. plana* while 56% of all observed females (*n* = 1222) were foraging on *H. diversicolor*. This difference was apparent regardless of the predominant prey type being consumed by the majority of individuals in the flock, with male godwits consuming proportionately more *S. plana* and females taking proportionately more *H. diversicolor* (Fig. 3).

**Figure 4:**
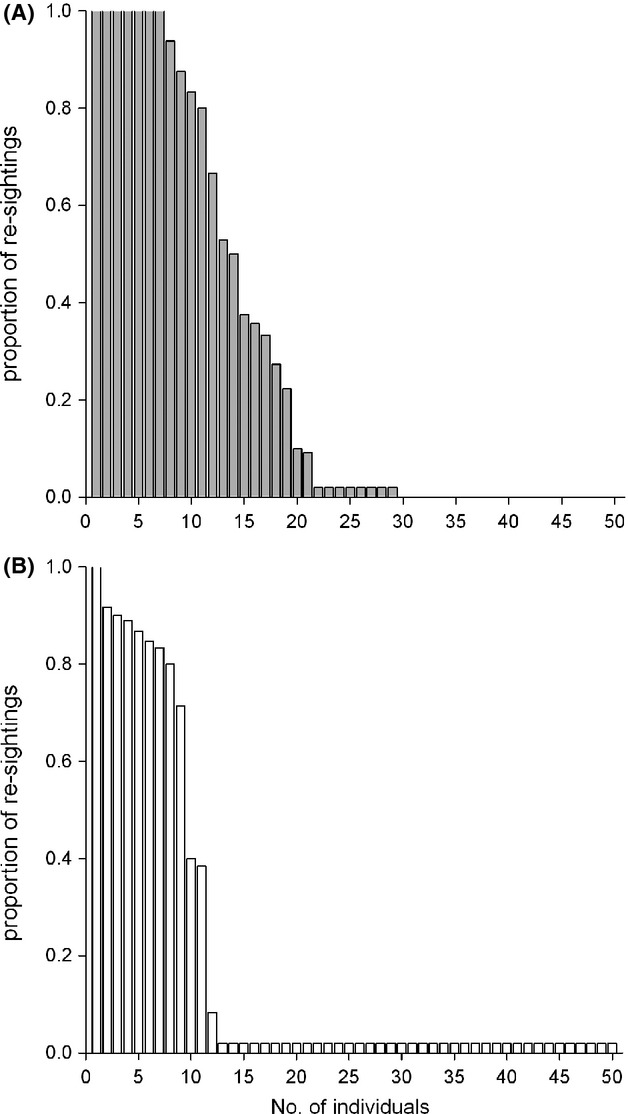
Proportion of re-sightings of each individually color-ringed (A) female (*n* = 29) and (B) male (*n* = 50) godwit on sites dominated by *Hediste diversicolor* during the winter of 2007–2008 (sites 3 and 6 on Fig. 2).

Using morphological characteristics to assign sex to color-ringed individuals of known sex indicated that males were correctly assigned on 86% ± 1.6 SE of all observations (*n* = 450) and females on 80% ± 3.6 SE of all observations (*n* = 227). The locations of color-ringed females (*n* = 29) and males (*n* = 50) were recorded during fortnightly surveys of the estuarine sites used by godwits across the Tagus estuary, with each godwit being recorded at least once every month (mean number of observations per individual = 11.8 ± 0.4 SE, range = 6–22). In order to explore whether the inclusion of incorrectly sexed individuals may have influenced the observed sex differences in use of prey resources, the use of sites dominated by *H. diversicolor* (sites 3 and 6, Fig 2) was compared among color-ringed individuals of known sex (Fig. 4). Of 29 color-ringed females, 21 were recorded on sites dominated by *H. diversicolor* and the average proportion of re-sightings of marked females on these sites was 48% ± 7.7 SE. By contrast, of 50 color-marked males, only 12 were seen on sites dominated by *H. diversicolor*, and the average proportion of re-sightings of male godwits on these sites was significantly lower (17% ± 3.4 SE; W = 374.5, df = 77 *P* < 0.001). Moreover, only 24% of the males were ever recorded in *H. diversicolor*-dominated sites, while only 29% of the females were never recorded in these sites (Fig. 4).

### Sex-specific prey consumption and prey profitability

**Figure 5:**
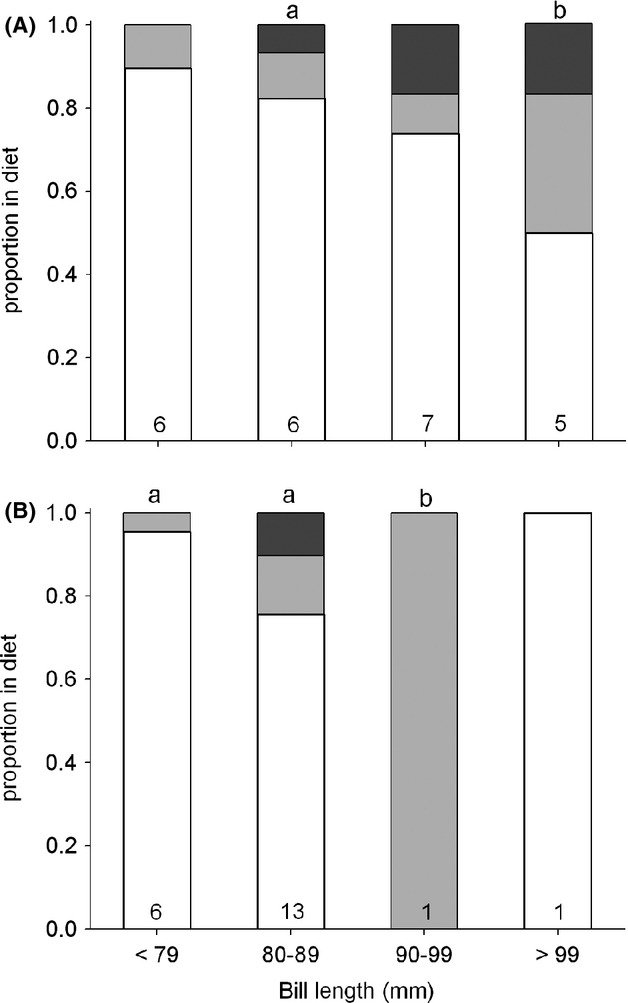
Size distribution of different size classes (white – small and medium, gray – large, and black – very large) of (A) *Hediste diversicolor* or (B) *Scrobicularia plana* consumed by individually color-ringed black-tailed godwits with different bill lengths. Numbers of individual godwits in each category are shown within the bars and bill length categories also indicate sex: males <89 mm and females >90 mm. Different letters indicate significant differences (*P* < 0.05) from Fisher exact tests of independence between each bill length category.

The distribution of prey sizes consumed by individual godwits varies in relation to bill length (Fig. 5). For color-ringed birds of known bill lengths, those with very short bills (<79 mm) consumed mostly (>90%) small and medium polychaetes and bivalves and were never recorded consuming very large prey. For godwits with larger bills, the proportion of large and very large *H. diversicolor* increased with bill size and, of the polychaetes consumed by females with the largest bills (>99 mm), ca. 50% are large or very large (Fig. 5A). Males with bill lengths of 80–89 mm consume larger and very large *S. plana* than smaller billed males, but only two color-ringed females were ever recorded foraging on bivalves (Fig. 5B).

**Figure 6:**
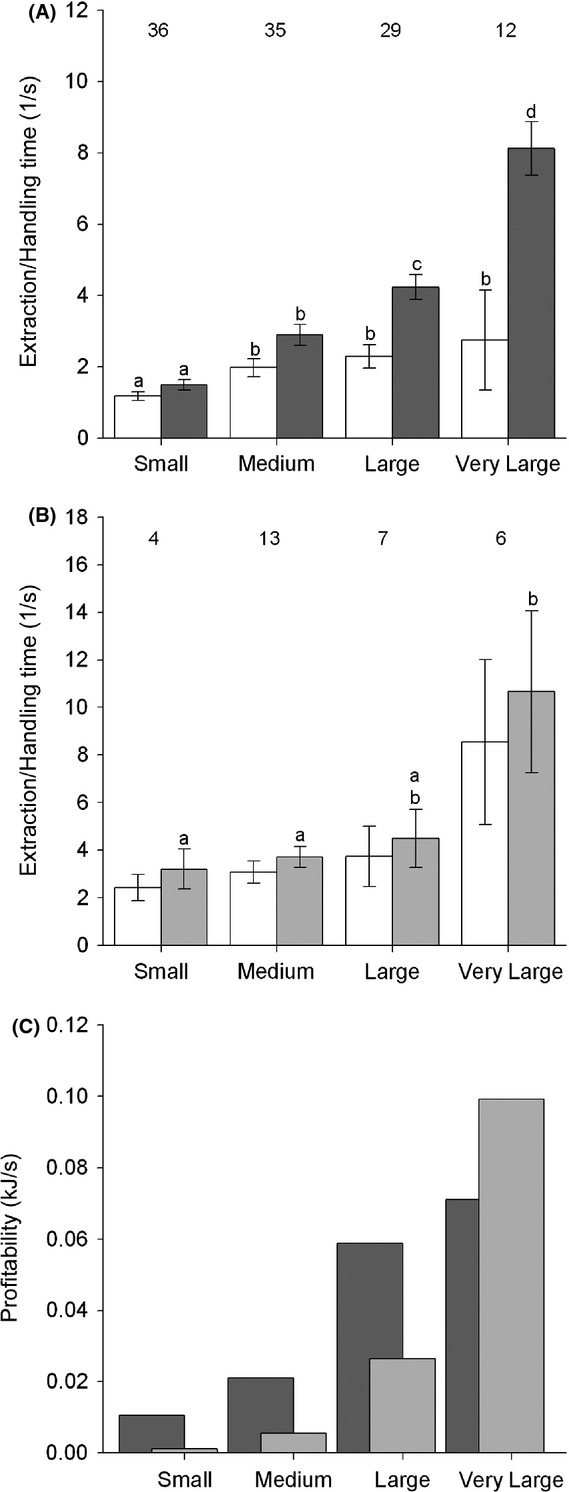
Differences in mean (± SE) handling time (gray bars) and extraction time (white bars) for black-tailed godwits foraging on four prey size classes of (A) *Scrobicularia plana* and (B) *Hediste diversicolor*; and (C) the estimated profitability (or handling efficiency, kJ/s^1^) of each size class of *Scrobicularia plana* (dark gray bars) and *Hediste diversicolor* (light gray bars). Numbers above bars indicate sample sizes and different letters indicate significant differences (*P* < 0.05) from post-hoc tests.

For godwits foraging on *S. plana*, both prey extraction times and prey handling times increase significantly with prey size (extraction time: F_3,107_ = 5.21, *P* = 0.002, handling time: F_3,107_ = 51.39, *P* < 0.001, Fig. 6A). For godwits foraging on *H. diversicolor*, extraction and handling times also increase with prey size, but these differences are only statistically significant for handling times (extraction time: F_3,26_ = 2.69, *P* = 0.07, handling time: F_3,26_ = 4.41, *P* = 0.01, Fig. 6B). The estimated profitability of *S. plana* is higher than *H. diversicolor* for small, medium, and large prey sizes; however, very large *H. diversicolor* are approximately 28% more profitable than very large *S. plana* (Fig. 6C).

## Discussion

### Sexual segregation in Icelandic godwits

For migratory sexually size-dimorphic species, the costs associated with different body sizes could result in the sexes wintering in different locations and/or using different resources, as a consequence of processes such as size-specific dominance, predation risk or access to differing resources (Durrell et al. 1993; Zharikov and Skilleter 2002; Nebel 2005; Mathot et al. 2007). In Icelandic godwits, there is no clear large-scale sexual segregation, but small-scale sex differences in the use of estuarine prey resources are apparent. Within the Tagus estuary, male and female godwits show significant differences in resource preference and spatial use of the estuary linked to resource distribution. Male godwits forage mostly on *S. plana* and, even among flocks foraging primarily on *H. diversicolor*, males are more likely than females to consume bivalves. By contrast, females are more likely than males to forage on polychaetes, irrespective of the prey type being consumed by the majority of the flock. Sex differences in prey preference are also indicated by the distinct spatial distribution of individually marked godwits, with females being recorded frequently in areas dominated by *H. diversicolor,* but very few males being recorded on these sites (Fig. 4). The fact that females prefer *H. diversicolor* and are more frequently recorded in sites dominated by this prey is likely to be linked to accessibility of this prey type. As large *H. diversicolor* burrow deeper in the sediment (individuals exceeding 100-mm body length typically burrow to depths of ca. 90–120 mm, Esselink and Zwarts 1989), large polychaetes may only be accessible to individuals with longer bills (Fig. 5A). These prey accessibility limitations do not apply to *S. plana*, as the shallower burying depth of all the size classes consumed by godwits (<20 mm shell length) will be within reach of even the smallest godwit bill (Zwarts et al. 1996). Sex differences in bill size have also been related to dietary differences in oystercatchers (*Haematopus ostralegus*) (Durrell et al. 1993), bar-tailed godwits (Smith and Evans 1973; Zwarts 1985), and western sandpipers (Nebel 2005; Mathot et al. 2007). Small-scale sexual segregation of dimorphic shorebirds may therefore be widespread, and is likely to be a consequence of the differences in the spatial distribution of different prey types within estuaries.

### Energetic implications of sex differences in resource use

Sexual dimorphism in Icelandic godwits results in the females (the larger sex) experiencing higher food demands, and Icelandic godwits wintering in Portugal require an additional 1390 kJ to complete their migration to Iceland than conspecifics wintering in England or Ireland (Alves et al. 2013). However, a larger body mass also provides a wider range of thermoneutrality (McNab 1970, 1980) and the living costs for Icelandic godwits are up to 38.4 kJ/day higher in the northern winter sites. However, the difference in energetic demand (i.e., maintenance metabolism) between the sexes is negligible (Alves et al. 2013). The mild climatic conditions on the Tagus estuary mean that neither male nor female black-tailed godwits need to thermoregulate during winter (Alves et al. 2013), hence no extra energy is needed to maintain core body temperature for either sex. Thus, for the smaller bodied (and smaller billed) godwit males that might be less capable of accessing large, deeper burrowed polychaetes, foraging on small, medium or large *S. plana* is more profitable than foraging on polychaetes of these size classes (Fig. 6). Male godwits do indeed forage preferentially on *S. plana* (Fig. 3) and the increasing proportion of large prey with bill length, independent of sex, suggests that this is largely a function of their smaller body size. However, large females are able to include a high proportion of the most profitable larger *H. diversicolor* size classes in their diet (Fig. 5A) and are therefore likely to meet their energetic requirements within a shorter foraging period than males. The Tagus estuary is the most southerly of the major godwit wintering locations (Alves et al. 2010), and very large *H. diversicolor* are proportionately much more abundant on the Tagus (∼25% of all polychaetes sampled) than on more northerly estuaries within the range (east England estuaries: ∼4%, Gill et al. 2001b; south Ireland estuaries ∼1%, Hayhow 2009). The capacity of large female godwits to migrate to the south of the range may thus be facilitated by the abundance of large polychaetes at this site.

### Implications of sexual segregation for migratory shorebirds

Male and female Icelandic godwits show distinct prey preferences at the estuary-scale, but do not display a clear pattern of large-scale sexual segregation across the winter range, probably because the distribution of the two main prey types varies little across this range (Piersma et al. 1993). The fact that the overall sex ratio on the Tagus is unbiased (Fig. 1), even though small-scale sexual segregation within this estuary is apparent, indicates that local-scale variation in prey distribution is likely to be the main driver of the scale of sexual segregation. Large-scale sexual segregation linked to prey distribution has been reported in other shorebird species (Mathot et al. 2007), and may have potential demographic consequences if, for example, different areas of a winter range vary in their exposure to extreme environmental conditions, which could then disproportionately affect one of the sexes (Cristol et al. 1999; Durell 2000). Higher rates of female mortality have recently been reported for many avian species (Donald 2007) and large-scale sexual segregation or sexual bias could contribute to such differences, particularly in species in which females have greater energetic requirements and migration costs (Alves et al. 2012a). Sex-specific predation risk has also been indicated as a contributing factor to sexual segregation in western sandpipers, with females (the larger sex) potentially avoiding northerly wintering sites where the need to carry more fat disproportionately increases their predation risk (Nebel and Ydenberg 2005). However, both male and female Icelandic godwits show no significant variation in body mass throughout their relatively small winter range (Alves et al. 2012a), and there is no evidence of large-scale variation in predation risk. The costs and benefits of different body sizes can also vary between seasons. In the breeding season, smaller male godwits tend to be more abundant on better quality sites where breeding success is higher, and there is evidence for larger females tending to mate with smaller males (Gunnarsson et al. 2012). Thus, body size distribution and the fitness implications for different phenotypes are likely to be shaped by these trade-offs and seasonal interactions.

Estuarine invertebrate populations are subject to a wide range of anthropogenic impacts. On many estuaries through Europe, extensive shellfishing (Piersma et al. 2001; Van Gils et al. 2006) and wastewater discharges have significantly influenced the structure and abundance of invertebrate communities (Beukema 1991; Savage et al. 2002). On the Tagus estuary, the abundance of very large polychaetes is related to high level of wastewater discharge (Alves et al. 2012b). The effects of these changes on species with small-scale sexual segregation in resource use might differ for males and females, with potential implications for sex-biased mortality, although the impact will depend on the magnitude of the changes. The apparent shift toward polychaete-dominated communities in some major European estuaries (Reise 1982; Piersma et al. 2001; Philippart et al. 2007; Atkinson et al. 2010) might have disproportionate impacts on males in species in which larger females have a greater capacity to access deeply buried polychaetes. However, ongoing efforts in Europe to reduce wastewater inputs into estuaries can also influence the abundance of polychaetes (Ait Alla et al. 2006; Alves et al. 2012b), which may disproportionately influence females of these species, particularly if they are important for fuelling long migratory journeys. Consequently, small-scale sexual segregation in resource availability and use can result in disproportionate effects of environmental change on males and females, and the implications of such changes are likely to vary in relation to the migratory distances and costs experienced by individuals.
